# The Influence of Increased Body Fat or Lean Body Mass on Aerobic Performance

**DOI:** 10.1371/journal.pone.0095797

**Published:** 2014-04-21

**Authors:** Marcin Maciejczyk, Magdalena Więcek, Jadwiga Szymura, Zbigniew Szyguła, Szczepan Wiecha, Jerzy Cempla

**Affiliations:** 1 Institute of Biomedical Sciences, Department of Physiology and Biochemistry, University School of Physical Education, Krakow, Poland; 2 Department of Clinical Rehabilitation, University School of Physical Education, Krakow, Poland; 3 Institute of Biomedical Sciences, Department of Sport Medicine and Human Nutrition, University School of Physical Education, Krakow, Poland; University of Barcelona, Faculty of Biology, Spain

## Abstract

**Purpose:**

The purpose of this study was to determine aerobic performance in men with an increased body mass due to (a) high body fat (>21.5%) but with a average (59.0–64.3 kg) lean body mass (HBF group) and (b) high lean body mass (>66.3 kg), but with average body fat (14.0–18.5%) (HLBM group).

**Methods:**

The men in the HBF and HLBM had similar absolute body mass and body mass index (BMI). The aerobic performance was also determined in control group. Methods: Study participants comprised 39 men aged 21.3±1.9 years who did not participate in competitive sports but were recreationally physically active. Participants were divided into three groups. Each group comprised 13 persons. The study involved anthropometric measurements, assessing aerobic performance (VO_2_max) using an incremental test on a mechanical treadmill. VO_2_max was expressed in absolute values, relative to body mass (VO_2_max⋅BM^−1^), relative to lean body mass (VO_2_max⋅LBM^−1^), and relative to BM raised by the exponents of 0.75 and 0.67. Body composition was measured using bioelectrical impedance analysis.

**Results:**

No statistically significant differences in relative values of VO_2_max were found between the HBF and HLBM groups, in VO_2_max⋅BM^−1^ (50.24±4.56 vs. 53.11±5.45 mL⋅kg^−1^), VO_2_max⋅LBM^−1^ (65.33±5.63 vs. 63.86±7.13 mL⋅kgLBM^−1^), and VO_2_max⋅BM^−0.75^ (150.29±13.5 vs. 160.39±16.15 mL⋅kg^−0.75^). Values of VO_2_max⋅BM^−1^ were significantly lower in the HBF and HLBM groups than in the control group (58.23±5.84 mL⋅kg^−1^).

**Conclusion:**

High body mass, regardless of the cause decreases VO_2_max⋅BM^−1^.

## Introduction

Aerobic exercise performance is indicated by maximal oxygen uptake per minute (VO_2_max) and primarily determined by the efficiency of mechanisms supplying active muscles with oxygen from the air [1]. Other factors affecting aerobic performance include body mass (BM) and body composition [2]. Obese and overweight persons, whose high BM is caused by high body adiposity, display a considerably lower VO_2_max relative to their body mass [3], [4]. However, a high body mass, as well as a high body mass index (BMI), can also be caused by a high amount of lean body mass (LBM) in persons with normal (or even low) body fat (BF). Publications to date have presented results of research on the influence of obesity and overweight on physical fitness and have established correlations between body composition and performance on fitness tests for athletes engaged in different disciplines [5], [6]. However, no attempts have thus far been made to conduct a comprehensive analysis of the influence of body composition on aerobic performance. The influence of body composition may be particularly important for sports disciplines in which athletes are required to have an appropriately high aerobic performance together with high muscle mass (e.g., boxing, basketball, or handball).

Traditionally, VO_2_max is given in absolute values and relative to BM. However, such method of data normalization does not account for body size and body composition. Darveau et al. [7] and West et al. [8] indicated a need to use parameters that allow for a comparison of physiological variables, such as VO_2_max, between persons with different BM. An example of such a parameter is the allometric scale [9], [10]. In relation to the practice of sports, studies have reported the need to use different values, such as allometric coefficients, to determine the percentage of total BM to be considered [11]. These values would be specific for each sport [10]. For runners, researchers have suggested normalizing results by providing oxygen uptake in mL^.^kg^−0.75.^min^−1^ [12], [13]. Most commonly, the two exponents of BM used as possible scaling factors are 0.67 and 0.75 [14].

Our hypothesis states that one’s endurance is affected by absolute BM regardless of body adiposity or LBM. Therefore, the main aim of this study was to determine aerobic performance in men with lower body mass and normal body composition and in men with an increased body mass due to (a) high body fat (but with a normal lean body mass) and (b) high lean body mass (but with normal body fat). The aims of the study also include determining the optimal method of expressing VO_2_max that would allow for a comparison of endurance between persons with different body mass and body composition.

## Methods

The study project was approved by the Commission for Bioethics at the Regional Medical Chamber in Krakow (opinion No. 88/KBL/OIL/2010) and procedures were carried out in accordance with Helsinki Declaration. Each study participant, having been informed of the aim and method of the study, signed an informed consent form to take part in the studies.

Before the incremental fitness test, each participant underwent a medical examination to ensure there were no contraindications to perform maximal physical effort. Anthropometric measurements and the incremental test were conducted before noon in similar external conditions (humidity and ambient temperature). Prior to the somatic measurements and the incremental test, participants were familiarized with the laboratory, measurement equipment, and testing procedures, and were instructed on how to prepare for the somatic measurements and the incremental test. Twenty four hours prior to testing participants were asked to refrain from physical activity, maintain hydration levels, and get at least 6 to 8 hours of sleep. Participants were also asked to consume a light meal at least 2 hours before testing.

### Somatic Measurements

The following parameters were determined: BM, LBM, BMI and body fat percentage (%BF). BH was measured using an anthropometer with 1 mm accuracy. Body mass was also raised to the 0.75 and 0.67 exponents (BM^0.75^ and BM^0.67^) [14]. BM and body composition was assessed by means of bioelectrical impedance analysis [15], using the Jawon IOI-353 Body Composition Analyzer (Korea; 8 electrodes, 3 measurement frequencies, tetra-polar electrode method). Body composition was assessed at normal body hydration (euhydration) in similar external temperature (22–24°C) [16]. Hands and feet were cleaned with alcohol before electrodes were placed on skin surface. The method used bioelectrical impedance which shows a high correlation (R = 0.88) with dual X-ray absorbtiometry [15].

### Division into Groups and Inclusion Criteria for each Group

Study participants had to meet specific body composition criteria. In preliminary research, anthropometric measurements were performed on 1,549 men aged 18–30 years (most of them were aged 19–23 years) to determine inclusion criteria for each group. For each body composition parameter, a measurement result between the 40th and 60th percentile within a given group was considered average; a result above the 80th percentile was considered high. Participants were divided into three groups of different body composition. Group 1, which was the control group, included men with mean %BF (14.0–18.5%) and mean LBM (59.0–64.3 kg). Group 2 included men with high %BF (>21.5%) and mean LBM (the High Body Fat [HBF] group). Group 3 included men with mean %BF and high (>66.3 kg) LBM (the High Lean Body Mass [HLBM] group). In addition, the men in the HBF and HLBM had BM values that were not significantly different (about 80–83 kg). Participants selected for the incremental test were comprised of men who took part in the introductory anthropometric assessment and met inclusion criteria related to BM and body composition (according to the division into groups).

### Participants

Ultimately, study participants comprised 39 physically fit college aged men (13 in each group), who agreed to take part in the assessment, met the aforementioned inclusion criteria, and did not participate in competitive sports but were physically active. Participants in all groups were of similar age (Table 1). The HBF group showed a statistically higher BM (due to high %BF) in comparison to the control group, but similar LBM and statistically higher BMI. The HLBM group showed statistically higher BM (due to high LBM) and similar %BF compared to the control group. BMI values in the HLBM group were also higher than in the control group. Statistically significant differences were observed in BH, %BF, and LBM between the HBF and HLBM groups. Table 1 shows detailed body composition parameters.

**Table 1:** Average (means±SD) age, body height, body mass, and body composition: lean body mass, body fat, and body mass index of study participants in each group.

Variables	Groups			Difference between groups		
	1: Control	2: HBF	3: HLBM	d2-1	d3-1	d3-2
N	13	13	13	–	–	–
Age (years)	21.2±1.4	21.6±2.9	21.2±1.3	0.4	0.0	–0.4
BH (cm)	179.0±3.7	177.8±3.5	185.2±5.2	–1.2	6.3*	7.4*
BM (kg)	73.3±2.3	80.2±4.4	83.3±3.8	6.9*	10.0*	3.1
LBM (kg)	61.4±1.3	61.6±2.7	69.4±4.0	−0.2	8.0*	7.8*
BF (%)	16.3±1.6	23.1±1.9	16.7±2.4	6.8*	0.4	−6.4*
BMI	22.9±1.3	25.4±1.1	24.4±1.5	2.5*	1.5*	−1.0

BH: body height, BM: body mass, LBM: lean body mass, BF: body fat, BMI: body mass index, *p<0.05.

### Assessment of Participants’ Physical Activity

Study participants did not engage in competitive sports but took part in recreational sports. For these reasons, the participants’ physical activity was assessed using a Seven Days Physical Activity Recall (7-day PAR) questionnaire [17], [18]. Before the interview, the participants were instructed on how to complete the questionnaire. They were asked to record physical activity they engaged in during the week before the incremental test. The men rated the intensity of their physical activity using three categories: moderate (e.g., a quick walk), hard (e.g., a slow run), and very hard (e.g., a brisk run or strength exercises).

Study participants differed considerably between groups in terms of duration and intensity of physical activity they undertook during the week. The HBF group was the least physically active, while the control and HLBM groups spent a similar amount of time on physical activity during the week but differed in terms of intensity. The control group declared engagement in significantly more (p<0.05) moderate-intensity exercises (in hours/week) than the HBF and HLBM groups (10.5±4.84 hr/week vs. 5.4±2.01 and 6.3±2.46 hr/week, respectively). The control and HLBM groups spent a similar amount of time during the week on hard-intensity exercises (3.0±2.13 and 2.6±1.35 hr/week, respectively), which was significantly greater than in the HBF group (1.0±0.42 hr/week). The HLBM group spent the most time on very hard–intensity exercises (including strength exercises), compared to 1.0±0.50 hr/week in the control group and only 0.5±0.57 hr/week in the HBF group.

### Incremental Test

The incremental exercise test was conducted on a Saturn h-p Cosmos treadmill (Germany). Physical effort of the participant began with a 4-minute warm-up at a speed of 7.0 km^.^h^−1^. Next, running speed was increased by 1.2 km^.^h^−1^ every 2 minutes until the participant reported extreme exhaustion and refused to continue the test. Oxygen uptake per minute (VO_2_) and pulmonary ventilation (V_E_) were measured during the test using a Medikro 919 M9427 ergospirometer (Finland). Heart rate (HR) was registered using a Polar S610i pulsometer (Finland). VO_2_max was considered equal to the value of VO_2_ that did not increase any further despite an increase in running speed or, in the case of the participant refusing to continue the test, equal to the highest registered value of VO_2_. VO_2_max was expressed in absolute values (L^.^min^−1^), relative to BM (VO_2_max^.^BM^−1^), relative to LBM (VO_2_max^.^LBM^−1^), and relative to BM raised by the exponents of 0.75 and 0.67 (VO_2_max^.^BM^−0.75^ and VO_2_max^.^BM^−0.67^) [14]. Additionally, testing time, running distance, and maximum running speed (v_max_) were measured for each participant.

### Biochemical Analysis

Venous blood samples were collected 5 minutes before and 3 minutes after the incremental test using Vacutainer BD blood collection equipment. Concentration of hydrogen ions (H^+^) was assessed each time after 2 ml of blood was collected with lithium heparin as an anticoagulant; a Siemens Rapid 348 analyzer (Germany) was used. Concentration of lactate anions (La^−^) was assessed by collecting 2 ml of blood into tubes with glycolysis inhibitors (5 mg of sodium fluoride and 4 mg of potassium oxalate). The blood was kept on ice no longer than 20 minutes and was centrifuged for 15 minutes at 4°C with RCF of 1.000×g. Immediately after centrifugation, 10 µl of blood plasma was taken and the concentration of La^−^ was measured using the L-Lactate Randox UK enzymatic test. Test sensitivity was 0.165 mmol^.^L^−1^, linearity was upwards of 19.7 mmol^.^L^−1^. Absorbency was measured at 550 nm using the UV/Vis Evolution 201 Thermo Scientific spectrophotometer (USA).

### Statistical Analysis

The one-factor ANOVA was used to determine differences in the assessed parameters among groups. The differences were assumed to be statistically significant for p<0.05. Next, post hoc comparisons were conducted using the Tukey’s HSD test to determine the significance of differences between mean values in a given group.

Pearson’s correlation coefficient (R) for correlations and coefficient of determination (R^2^) between selected dependent variables as well as VO_2_max (without the division into groups) were calculated to determine the optimal method of relativization of VO_2_max values. A correlation was assumed to be statistically significant for p<0.05 Statistica 8.0 (StatSoft, Inc., USA) software was used for statistical analysis.

## Results

Testing time, running distance, HRmax, v_max_, V_E_max, and absolute value of VO_2_max were similar for all men in the three groups (Table 2). The study also found no statistically significant differences between groups in concentrations of biochemical indicators (H^+^ and La^−^), as measured both before and 3 minutes after the test (Table 3). Post-effort La^−^ concentration amounted to about 12–13 mmol^.^L^−1^ (Table 3), which indicates that all groups achieved similar maximal intensity of effort toward the end of the incremental test. Values of VO_2_max relative to BM were significantly lower in the HBF and HLBM groups than in the control group. Values of VO_2_max relative to BM raised by the exponents of 0.75 and 0.67 were significantly lower in the HBF group than in the control group. No statistically significant differences in relative values of VO_2_max were found between the HBF and HLBM groups, i.e., in VO_2_max^.^BM^−1^, VO_2_max^.^LBM^−1^, VO_2_max^.^BM^−0.75^, and VO_2_max^.^BM^−0.67^ (Table 2).

**Table 2:** Running distance and time, maximum running speed, and maximum values of assessed physiological parameters: heart rate, pulmonary ventilation, and maximal oxygen uptake of study participants in each group obtained during the incremental test.

Variables	Groups			Difference between groups		
	1: Control	2: HBF	3: HLBM	d2-1	d3-1	d3-2
Distance (m)	3203±519	2930±560	3282±664	−273	79	352
v_max_(km^.^h^−1^)	15.37±1.15	14.78±1.37	15.50±1.49	−0.59	0.13	0.78
t (min:s)	17∶57±01∶57	16∶53±02∶15	18∶12±02∶30	−01∶04	00∶15	01∶19
HRmax (b^.^min^−1^)	204±7	200±9	199±8	−4	−5	−1
V_E_max (L^.^min^−1^)	150.44±19.61	143.83±20.12	158.67±15.36	−6.61	8.23	14.84
VO_2_max (L^.^min^−1^)	4.27±0.46	4.02±0.39	4.42±0.46	−0.25	0.15	0.4
VO_2_max^.^BM^−1^ (mL^.^kg^−1^)	58.23±5.84	50.24±4.56	53.11±5.45	−7.99*	−5.12*	2.87
VO_2_max^.^LBM^−1^ (mL^.^kg^−1^)	69.56±6.97	65.33±5.63	63.86±7.13	−4.23	−5.7	−1.47
VO_2_max^.^BM^−0.75^ (mL^.^kg^−0.75^)	170.40±17.28	150.29±13.5	160.39±16.15	−20.11*	−10.01	10.1
VO_2_max^.^BM^−0.67^ (mL^.^kg^−0.67^)	240.28±24.49	213.41±19.17	228.45±22.93	−26.87*	−11.83	15.04

v_max_: maximum running speed, t: time, HRmax: maximum heart rate, VO_2_max: maximal oxygen uptake, V_E_max: maximal pulmonary ventilation, BM: body mass, LBM: lean body mass, *p<0.05. Data are presented as means±SD.

**Table 3:** Average concentration of hydrogen ions and lactate anions in participants in each group before and after the incremental test.

Variables	Measurement	Groups			Difference between groups		
		1: Control	2: HBF	3: HLBM	d2-1	d3-1	d3-2
H^+^ (nmol^.^L^−1^)	before	39.66±2.39	40.55±3.73	39.70±2.05	0.89	0.04	−0.85
	after	60.70±9.01	57.53±8.73	61.83±7.77	−3.17	1.13	4.3
La^−^ (mmol^.^L^−1^)	before	1.22±0.61	1.31±0.83	1.35±0.86	0.09	0.13	0.04
	after	12.19±3.08	13.09±3.10	12.43±2.28	0.9	0.24	−0.66

H^+^: hydrogen ions; La^−^: lactate anions. Data are presented as means±SD.

Analysis of correlation coefficients between body composition parameters and VO_2_max showed several statistically significant correlations. A statistically significant positive correlation (R = 0.38) was found between LBM and absolute values of VO_2_max. Furthermore, a negative correlation was found between BM, BMI, and %BF and VO_2_max relative to BM. Statistically significant negative correlations were also found between BMI and VO_2_max^.^BM^−0.75^ or VO_2_max^.^BM^−0.67^ and between %BF and VO_2_max^.^BM^−0.75^ or VO_2_max^.^BM^−0.67^. VO_2_max^.^LBM^−1^ was the only parameter that showed no statistically significant correlation with body composition (Table 4).

**Table 4:** Pearson’s correlation coefficients R and coefficients of determination R^2^ [in square parentheses] between maximal oxygen uptake and selected parameters of body composition: body mass, body fat, and lean body mass.

	VO_2_max(L⋅min^−1^)	VO_2_max⋅BM^−1^(mL⋅kg^−1^)	VO_2_max⋅LBM^−1^(mL⋅kg^−1^)	VO_2_max⋅BM^−0.75^(mL⋅kg^−0.75^)	VO_2_max⋅BM^−0.67^(mL⋅kg^−0.67^)
BM (kg)	0.23 [0.05]	−0.39 [0.14]*	−0.29 [0.08]	−0.24 [0.06]	−0.20 [0.04]
BMI	−0.09 [0.00]	−0.47 [0.22]*	−0.22 [0.05]	−0.39 [0.15]*	−0.36 [0.13]*
BF (%)	−0.23 [0.05]	−0.40 [0.16]*	0.00 [0.00]	−0.36 [0.14]*	−0.36 [0.13]*
LBM (kg)	0.38 [0.14]*	−0.12 [0.01]	−0.28 [0.08]	0.00 [0.00]	0.04 [0.00]

VO_2_max: maximal oxygen uptake, BM: body mass, BF: body fat, LBM: lean body mass, *p<0.05.

## Discussion

The aim of the study was to determine the influence of body composition and increased BM on aerobic performance. High body mass can be caused by an increased amount of BF or increased muscle mass (i.e., LBM), or both. The study sought to isolate the influence of both factors. The study also sought to assess, on the one hand, the influence of high body adiposity and, on the other hand, the influence of high LBM in men with similar body mass and normal values of other parameters for body composition (with the exception of body height). Results show that most of the analyzed physiological and biochemical parameters were similar between the HBF and HLBM groups.

A statistically significant positive correlation was found between LBM and absolute values of VO_2_max. This correlation has been confirmed by other studies, which have found that high muscle mass (i.e., the main component of LBM) resulted in increased VO_2_ [3], [19]. McInnis and Balady [20], when comparing VO_2_ during submaximal effort between body builders (%BF = 8%) and men with normal body fat percentage (%BF = 24%) but having similar BM, found that body builders had a significantly higher VO_2_ during motor tasks. Therefore, high body mass does not limit the VO_2_max: no significantly lower absolute values of VO_2_max were found in the analyzed groups with high body mass in comparison to the controls. This study found similar absolute values of VO_2_max between the studied groups. This result is most likely due to the type and intensity of physical activity the study participants engaged in. Apart from body size and body composition, VO_2_max is determined by genetic factors as well as the type and duration of physical activity. The HBF group was the least physically active, while the HLBM group declared the greatest engagement in very hard–intensity exercises, including strength exercises. Conversely, the control group declared the greatest engagement in moderate-intensity exercises. Different intensity of exercises and the amount of time spent on physical activity between the groups may have affected the absolute values of VO_2_max. Low (although significant) correlation between absolute VO_2_max and LBM indicates the importance of the training state.

A problem that usually hampers interpretation of the data in a study of people with increased body mass and different body composition is the way of expressing the VO_2_max values. Depending on the chosen method of data normalization, results may be presented differently. People with similar absolute VO_2_max may have significantly different VO_2_max, which is relative to BM, yet at the same time, may have similar VO_2_max, which is relative to LBM. This study shows that the method of normalization of VO_2_max values is the determining factor in the interpretation of results of studies analyzing aerobic performance in persons with different body composition [21]. Various studies have analyzed different methods of normalizing VO_2_max values [10], [12], [13], [22]–[26].

The traditional and most commonly used method of expressing results of aerobic performance measurements is providing VO_2_max values relative to total body mass. In studies where VO_2_max values are expressed in this way, the results unambiguously show that high BM, regardless of body composition, has a negative effect on aerobic performance (the results of correlation in this study support this statement). A negative correlation between BM and VO_2_max^.^BM^−1^ suggests that persons with high BM have lower aerobic performance [24], [26], [27]. When the results were expressed in this way, the values of VO_2_max in the HLBM and HBF groups were similar (but significantly lower compared to the control group). However, Heil [22] showed that persons with low body mass were more likely to be categorized as having a low VO_2_max. Therefore, for two persons with similar LBM and similar absolute values of VO_2_max, if VO_2_ is expressed relative to BM, the person with lower body adiposity will display a higher aerobic performance.

The allometric scale allows for an analysis of physiological variables of specific groups while taking into consideration their characteristics, such as body composition, surface area of the body, level of training, and the environment of an activity [11], [28]. Studies have shown that different allometric exponents should be used for athletes from different sports disciplines [9]. Participants of this study did not engage in competitive sports and their physical activity differed in intensity, duration, and type of exercise. For these reasons, data analysis used the two most popular allometric exponents: 0.67 and 0.75. VO_2_max was expressed as power function ratios where, from the surface law, body mass should be raised to the power 0.67 [25], or, with acknowledgment of elasticity in body structures, 0.75 [12], [14]. This is to make the result of VO_2_max measurement independent of total body mass. The results of this study confirmed the validity of this approach: no correlation was found between BM and VO_2_max^.^BM^−0.75^ and VO_2_max^.^BM^−0.67^. However, there is a statistically significant negative correlation between VO_2_max^.^BM^−0.75^ and BMI and between VO_2_max^.^BM^−0.75^ and %BF (similar negative correlation was also noted between VO_2_max^.^BM^−0.67^ and %BF or BMI). When VO_2_max was expressed relative to BM^−0.75^ or BM^−0.67^, the HBF group displayed the lowest (p<0.05) values of this parameter.

Another suggested method of normalization is expressing VO_2_max relative to LBM [25], [27], [29]. In this study, when VO_2_max was expressed relative to LBM the all groups displayed similar results. At the same time, this method of normalization shows no significant correlation of VO_2_max with BM, body composition, and BMI. Therefore, this method seems to be optimal for comparing VO_2_max values in persons with different body composition.

The results of this study, which are similar to those found in studies of physical fitness in overweight or obese persons, demonstrated that obese persons displayed similar absolute VO_2_max values and VO_2_max values relative to LBM compared to persons with normal body composition [3], [30]. On the other hand, persons with high BF displayed significantly lower values of VO_2_max^.^BM^−1^ [3], [4]. Goran et al. [4] found that VO_2_max relative to LBM is an indicator of the physiological status of the cardio-respiratory system in terms of the oxidative demands of the body and does not seem to be influenced by excess FM. For this reason, it is recommended to provide VO_2_max relative to LBM, not relative to total BM [3]. The results of this study substantiate this recommendation: correlation coefficients indicate that body composition does not affect VO_2_max relative to LBM. However, there is a significant negative correlation between VO_2_max^.^BM^−1^ and %BF, BMI, and BM.

The limitation of the study was the significant difference in body height noted in HLBM compared to the control group and HBF groups. This is why the increased level of LBM may have been the result of the greater BH of persons in the HLBM group and not the result of, for example, the applied training. Nevertheless, tall persons were included in the study because adding another inclusion criterion would have considerably reduced the HLBM group. It should be noted that BH does not affect one’s endurance capabilities. VO_2_max is determined not only by somatic build, but also by many other factors, such as the cardiopulmonary functions, the number of erythrocytes, hemoglobin concentration in the blood, mass of the mitochondria, or the training state.

## Conclusions

Body mass shows a negative correlation with values of VO_2_max relative to body mass. This means that low values of this parameter are noted both in persons whose high body mass is the result of high body fat and in persons whose high body mass is the result of high lean body mass. Regardless of the method of normalization, aerobic performance of persons with similar body mass and different body composition is similar. High body mass, regardless of the cause (i.e., high BF or high LBM), decreases VO_2_max relative to body mass. Results of this study may prove useful for trainers, instructors, and persons engaged in anaerobic-aerobic sports disciplines that require good aerobic performance as well as strength and power determined primarily by muscle mass. The study found relatively low yet statistically significant correlation and determination coefficients that indicate a relationship between body mass and body composition and VO_2_max values expressed in the following ways: as absolute values relative to absolute body mass and as VO_2_max^.^BM^−0.75^ and VO_2_max^.^BM^−0.67^. VO_2_max expressed relative to LBM shows no relationship with absolute BM and body composition.
